# Impact of Gastric pH Variations on the Release of Amorphous Solid Dispersion Formulations Containing a Weakly Basic Drug and Enteric Polymers

**DOI:** 10.1021/acs.molpharmaceut.2c00895

**Published:** 2023-02-02

**Authors:** Hanh Thuy Nguyen, Tu Van Duong, Lynne S. Taylor

**Affiliations:** Department of Industrial and Physical Pharmacy, College of Pharmacy, Purdue University, West Lafayette, Indiana 47907, United States

**Keywords:** amorphous solid dispersion, delamanid, weakly basic drugs, enteric polymers, crystallization, gastric pH

## Abstract

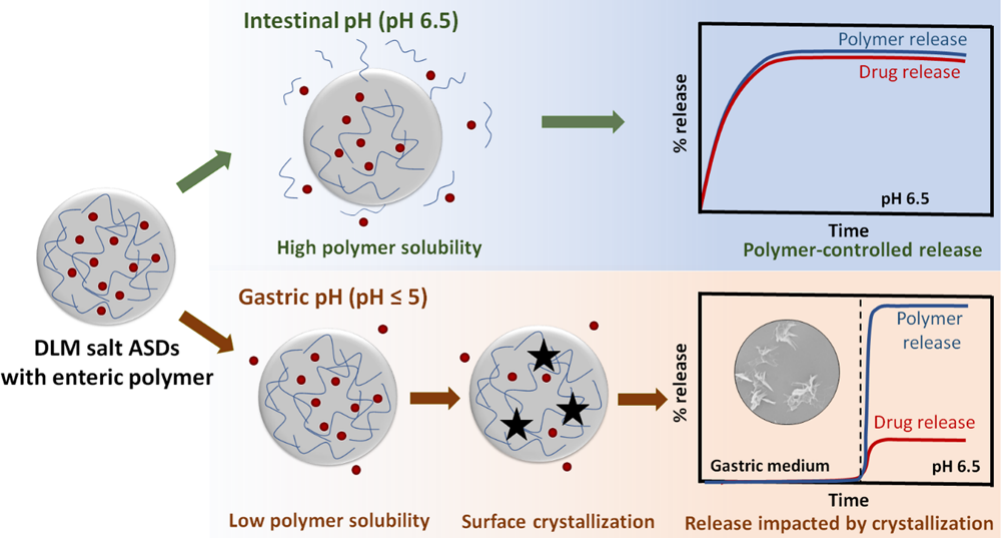

Enteric polymers are widely used in amorphous solid dispersion (ASD) formulations. The aim of the current study was to explore ASD failure mechanisms across a wide range of pH conditions that mimic in vivo gastric compartment variations where enteric polymers such as hydroxypropyl methylcellulose phthalate (HPMCP) and hydroxypropyl methylcellulose acetate succinate (HPMCAS) are largely insoluble. Delamanid (DLM), a weakly basic drug used to treat tuberculosis, was selected as the model compound. Both DLM free base and the edisylate salt were formulated with HPMCP, while DLM edisylate ASDs were also prepared with different grades of HPMCAS. Two-stage release testing was conducted with the gastric stage pH varied between pH 1.6 and 5.0, prior to transfer to intestinal conditions of pH 6.5. ASD particles were collected following suspension in the gastric compartment and evaluated using X-ray powder diffraction and scanning electron microscopy. Additional samples were also evaluated with polarized light microscopy. In general, ASDs with HPMCP showed improved overall release for all testing conditions, relative to ASDs with HPMCAS. ASDs with the edisylate salt likewise outperformed those with DLM free base. Impaired release for certain formulations at intestinal pH conditions was attributed to surface drug crystallization that initiated during suspension in the gastric compartment where the polymer is insoluble; crystallization appeared more extensive for HPMCAS ASDs. These findings suggest that gastric pH variations should be evaluated for ASD formulations containing weakly basic drugs and enteric polymers.

## 1 Introduction

In recent years, there has been an increase in the number of active pharmaceutical ingredients that have low solubility, leading to limited oral bioavailability. To address poor solubility and suboptimal dissolution, thereby improving oral absorption, formulation strategies such as amorphous solid dispersion (ASD) are typically employed. Molecular dispersion of a drug in a hydrophilic polymer can generate highly supersaturated drug solutions upon dissolution.^1,2^ However, supersaturated solutions are thermodynamically metastable/unstable and tend to undergo crystallization.^3^ Several polymers have been found to be effective crystallization inhibitors, stabilizing supersaturated solutions by inhibiting nucleation and crystal growth.^4,5^ Drug–polymer interactions may include π–π interactions,^6^ ionic interactions,^7^ and hydrogen^5,8^ or halogen bonding,^9^ with nonspecific interactions between hydrophobic groups thought to be important for crystallization inhibition in an aqueous environment.^10^ Moreover, polymers also need to have hydrophilic groups to promote hydration and facilitate drug release from the ASD.^10,11^

Amphiphilic, ionizable polymers, such as hydroxypropyl methylcellulose acetate succinate (HPMCAS), hydroxypropyl methylcellulose phthalate (HPMCP), Eudragit L100, and cellulose acetate phthalate, have been found to be effective at maintaining drug supersaturation and enhancing in vivo absorption.^4,7,11−13^ Among these, HPMCP and HPMCAS have been noted to be good crystallization inhibitors for many drugs.^6,14,15^ Several commercial products are prepared as ASDs with HPMCAS, including telaprevir (Incivek, Vertex, US); ivacaftor (Kalydeco, Vertex, US); vemurafenib (Zelboraf, Roche, Switzerland); posaconazole (Noxafil, Merck, US); and apalutamide (Erleada, Janssen, US).^16−20^ In contrast, HPMCP has been used less for commercial ASD formulations, with its use reported only for itraconazole (Lozanoc, Mayne Pharma, Australia)^16,18,21^ and delamanid (DLM) (Deltyba, Otsuka, Japan).^22^ Importantly, enteric polymers are un-ionized and insoluble in acidic environments, leading to reduced crystal growth inhibition effectiveness at low pH.^10,23−25^ For example, HPMCAS (-LF grade) was found to be less effective at inhibiting the crystal growth of felodipine at a lower pH than at a higher pH.^24^ Therefore, if a drug has a tendency to undergo crystallization when the formulation is immersed in the gastric fluid, the inhibitory properties of a polymer in this environment need to be considered.^6,26,27^

In addition, it may be more challenging to inhibit the crystallization of Biopharmaceutics Classification System class II weakly basic drugs due to their pH-dependent solubility.^28−30^ These compounds can undergo ionization and dissolution in acidic media but may crystallize upon transit to the higher pH environment of the small intestine.^27,28,31^ DLM, a nitroimidazo-oxazole developed by Otsuka Pharmaceutical Co., Ltd. (Tokyo, Japan), is a weakly basic drug with poor solubility and low bioavailability.^32^ Previous work in our group indicated that salt formation reduced the risk of drug crystallization during ASD manufacturing and storage.^15^ However, drug absorption has been found to be dependent on food intake and fed state dosing is recommended for DLM.^33−35^ In the presence of food, drug solubility and dissolution may be impacted by several factors such as pH, buffer capacity, surface tension, protein concentration, bile salt content, and lipid content.^36−39^ In particular, peak gastric pH may increase to ∼6.0–7.0 before decreasing to fasting levels after 1–4 h, depending on meal composition, amount, and pH.^40−42^ Generally, three pH values of 6.4, 5.0, and 3.0 are considered as representative media to simulate conditions for “early” (first 20–75 min), “middle” (until 160 min), and “late” (after 160 min) time points following meal ingestion.^43−45^ The solubility and crystallization behavior of DLM formulated as an ASD in solutions of low and intermediate acidity is unknown, but could have important consequences for drug release and absorption. This is an important consideration, given that DLM is formulated with an enteric polymer,^22^ whereby the formulation is nominally insoluble at low pH due to protonation of the polymer; however, at low pH, DLM is ionized and may leach from the formulation. Another issue noted for DLM free base is that the drug has a high tendency to crystallize during manufacturing and storage. Interestingly, salts with certain counterions, including edisylate, were found to have improved amorphous form physical stability.^15^ Furthermore, ASDs of DLM edisylate with enteric polymers resulted in a notable enhancement in the extent of drug release in phosphate buffer pH 6.5 as compared to the neat amorphous DLM edisylate material, as well as the free base ASD.^15^

The overall aim of this study was to evaluate the impact of different types and grades of ionizable polymers on the release profile and crystallization of DLM ASDs following two-stage release testing where the pH of the gastric compartment is varied to simulated fed and fasted conditions. We hypothesized that the pH-dependent drug crystallization tendency, the form of DLM in the ASD (free base versus edisylate salt), and drug/polymer solubility as a function of pH play important roles in determining the in vitro release profiles of DLM. ASDs of DLM free base or edisylate salt were prepared with HPMCP (P-50 and P-55 grade) and HPMCAS (-LF, -MF and -HF grade) by rotary evaporation or spray drying. Drug and polymer release from ASDs were evaluated in pH 6.5 buffer or in two-stage pH-shift release experiments. Drug crystallization was monitored using polarized light microscopy (PLM), X-ray powder diffraction (PXRD), and scanning electron microscopy (SEM).

## 2 Experimental Section

### 2.1 Materials

DLM was purchased from Gojira Fine Chemicals, LLC (Bedford Heights, OH). HPMCP (P-50 and P-55 grade) and HPMCAS (-LF, -MF and -HF grade) were provided by Shin-Etsu Chemical Co., Ltd. (Tokyo, Japan). 1,2-Ethanedisulfonic acid dihydrate was procured from Tokyo Chemical Industry Co. Ltd. (Tokyo, Japan). Sodium chloride, sodium hydroxide, sodium phosphate monobasic monohydrate, hydrochloric acid, and organic solvents, including dichloromethane (DCM), methanol (MeOH), dimethyl sulfoxide (DMSO), acetonitrile, and acetone, were obtained from Fisher Scientific (Pittsburg, PA).

### 2.2 Drug and Polymer pH-Dependent Solubility

#### 2.2.1 Drug Solubility

The equilibrium crystalline solubility of DLM in HCl solution pH 1.6 and phosphate buffer (pH range from 2.0 to 6.5, solution compositions are summarized in Table 1) was measured by adding an excess amount of the compound to 15 mL of medium and stirring at 37 °C, 300 rpm. After 48 h, 10 mL of solution was withdrawn and ultracentrifuged (SW 41Ti rotor, 35,000 rpm, 37 °C, 30 min) using an Optima L-100 XP ultracentrifuge (Beckman Coulter, Inc., Brea, CA). The drug concentration was determined by high performance liquid chromatography (HPLC, Agilent Technologies, Santa Clara, CA) with a C18 column (4.6 × 250 mm, 5 μm), mobile phase of acetonitrile-water (75:25 v/v) at a flow rate of 1.5 mL/min, and a UV detector of 320 nm. A calibration curve was built over the drug concentration range of 0.01–50 μg/mL.

**Table 1:** Composition of HCl Solution pH 1.6 and Phosphate Buffer Solutions with pH Ranging from 2.0 to 6.5^30^

pH of solution	buffer solution composition					
	H_3_PO_4_ (0.1 M, mL)	NaH_2_PO_4_ (0.14 M, mL)	NaOH (0.1 M, mL)	NaCl (mg)	HCl (0.2 M, mL)	water (mL)
1.6				400	20	180
2.0	108.0	100.0		833.9		
2.5	50.4	150.0		396.5		
3.0	21.6	200.0		171.0		
3.5	7.2	200.0		57.1		
4.0	2.0	200.0		15.9		
4.5		200.0	1.2			0.9
5.0		200.0	6.4			2.8
5.5		200.0	19.2			8.3
6.0		150.0	36.9			15.8
6.5		150.0	79.8			34.2

The amorphous solubility of the drug as a function of pH was determined by the UV extinction method^46^ using a 5 mm UV probe SI Photonics UV/vis spectrometer (Tucson, Arizona). A stock solution of DLM in DMSO was introduced into 50 mL of aqueous solution (HCl solution pH 1.6 or phosphate buffer with a pH over the range of 2.0–6.5) at a flow rate of 100 μL/min using a Harvard PHD 22/2000 syringe pump (Harvard Apparatus, Holliston, MA). The light scattering at non-absorbing wavelengths (450 nm) was used to determine the amorphous solubility.

#### 2.2.2 Polymer Solubility

Polymer solubility was also measured over the pH range of 1.6–6.5 using the same media as described above. 50 mg of HPMCP (P-50 and P-55) or HPMCAS (-LF, -MF, and -HF) was added to 15 mL of media. After stirring at 37 °C for 48 h, samples were centrifuged at 35,000 rpm, 37 °C for 30 min using an Optima L-100 XP ultracentrifuge with SW 41Ti rotor (Beckman Coulter, Inc., Brea, CA). The polymer concentration was quantified by an HPLC equipped with an evaporative light scattering (ELSD, Agilent, Wilmington, DE) detector. The polymer in the supernatant obtained after centrifugation was hydrolyzed by mixing 1 mL of supernatant with 0.5 mL of 2 M sodium hydroxide solution, followed by incubation overnight.^47^ The solution was then neutralized by adding 0.5 mL of 2 M hydrochloric acid solution and diluted with methanol to a concentration within the range of the calibration curve. The cellulose backbone concentration was evaluated using the chromatographic conditions described in Table S1. A calibration curve for each polymer was built over the concentration range of 5–400 μg/mL. In addition, colorimetric analysis^48^ was conducted to verify the results of the HPLC-ELSD method. Briefly, 10 μL of phenol was added to 400 μL of diluted sample (in phosphate buffer pH 6.5), followed by addition of 1 mL of concentrated sulfuric acid. The reaction of the cellulose backbone and phenol in the presence of sulfuric acid led to the formation of an orange-yellow color, which was determined by colorimetric analysis with a UV-1600 PC UV/vis spectrophotometer (VWR International, Radnor, PA) at 490 nm. A calibration curve for each polymer was prepared in the range of 1–100 μg/mL.

### 2.3 ASD Preparation

ASDs of the DLM edisylate salt with the enteric polymers were prepared in situ by rotary evaporation . The drug, 1,2-ethanedisulfonic acid, and polymer were dissolved in a mixture of acetone-DCM (1:1 v/v). Solvents were removed at 40 °C, 150 rpm using a Buchi Rotavapor-R (Newcastle, Delaware). ASDs were then kept in a vacuum oven overnight to remove residual solvent, followed by cryo-milling and sieving to obtain particles in the range of 106–250 μm. An ASD of DLM free base and HPMCP-50 was prepared by spray drying using a Buchi Mini Spray Dryer B-290 equipped with an Inert Loop B-295 (Buchi, New Castle, DE). Drug and polymer were dissolved in MeOH-DCM (1:1 v/v) at a solid content of 10% w/v. The spray drying process was conducted under a nitrogen stream at a flow rate of 700 L/h, aspirator 35 m^3^/h with a feed rate of 4 mL/min, and inlet temperature of 75 °C. Spray-dried ASDs were also secondary dried in the vacuum oven overnight before further analysis.

### 2.4 Release Studies

Release studies were performed with a tablet formulation of the ASD to circumvent wetting issues with the ASD powder. The tablet (75 mg) contained ASD powder (equivalent to 5 mg DLM) mixed with excipients (sodium starch glycolate, 4 mg; croscarmellose sodium, 4 mg; silica colloidal hydrate, 0.6 mg; magnesium stearate, 0.6 mg; Avicel PH 101, q.s. 75 mg).^15^ Release testing was conducted at 150 rpm, 37 °C, using a USP apparatus II (Hanson, Billerica, MA). Release studies were performed in a single-stage medium (phosphate buffer, pH 6.5) or using pH shift experiments, with the first dissolution stage at an acidic pH (0.02 M HCl solution, pH 1.6, or phosphate buffer at pH 3.0 or pH 5.0) for 60 min, followed by dissolution in phosphate buffer pH 6.5 for an additional 30 min. The low pH media were converted to pH 6.5 by adding concentrated phosphate buffer pH 7.3, the composition of which is presented in Table 2. The dose concentration in the dissolution medium was 100 μg/mL.

**Table 2:** Dissolution Media Compositions

buffer type	NaOH (g)	NaH_2_PO_4_.H_2_O (g)	NaCl (g)	water q.s. (mL)
concentrated buffer pH 7.3 (0.57 M)	17.4	79	11.1	1000

dissolution medium	*V* (mL) in the first 60 min	*V* (mL) buffer pH 7.3 to adjust to pH 6.5	final volume (mL)	final pH
0.02 MHCl, pH 1.6	45	5	50	6.4–6.6
phosphate buffer pH 3.0 (0.1 M)	47	3	50	6.4–6.6
phosphate buffer pH 5.0 (0.13 M)	48	2	50	6.4–6.6
phosphate buffer pH 6.5 (0.08 M)	50	0	50	6.5–6.6

Drug release as a function of time was determined using an in situ dissolution monitoring system (Pion Rainbow Instrument, Billerica, MA) based on fiber optic UV spectroscopy. Drug concentration was calculated from analysis of second derivative spectra by determining the area under the curve over the wavelength range of 330–350 nm. Calibration curves of DLM in different buffers were built over the concentration range of 1–100 μg/mL.

Polymer release was evaluated at different time points by withdrawing 1.5 mL of dissolution medium, which was replaced with fresh media, followed by centrifugation at 14,800 rpm, 37 °C for 3 min to remove undissolved ASDs. Polymer concentration was determined by the HPLC-ELSD method as described above.

### 2.5 Powder X-ray Diffraction

PXRD was used to confirm the amorphous state of freshly prepared DLM ASDs and to detect drug crystallization in solution. For the amorphous DLM edisylate, an excess amount of the drug salt was added to different media, including acidic fluids at pH 1.6, 3.0, or 5.0 and intestinal fluid pH 6.5 (compositions of which are described in Table 1), and kept stirring at 300 rpm, 37 °C. Crystallization of the DLM salt was evaluated at predetermined time points. Drug crystallization in ASDs upon acidic incubation was conducted at the same drug concentration as in the dissolution testing: 20 mg of ASD powder was added to 50 mL of acidic solutions at pH 1.6, 3.0, or 5.0 and kept stirring at 300 rpm, 37 °C for 1 h.

Undissolved particles were collected by vacuum filtration and analyzed using a Rigaku Smartlab diffractometer (Rigaku Americas, The Woodlands, TX) equipped with a Cu–Kα radiation source and a D/tex ultradetector. Diffractograms were acquired with a scanning speed of 4°/min (4–40° 2θ) and 0.02° step size. The voltage and current were 40 kV and 44 mA, respectively.

### 2.6 Polarized Light Microscopy

Drug crystallization during incubation in acidic media was also evaluated by PLM using a Nikon Eclipse E600 microscope coupled with a Nikon DS-Ri2 camera (Melville, NY). A thin film ASD was prepared by a spin-coater KW-4A (Chemat Technology Inc., Northridge, CA). A solution of drug, 1,2-ethanedisulfonic acid, and polymer in organic solvents (acetone-DCM 1:1 v/v) were dropped onto a square cover glass (22 mm × 22 mm) and kept spinning at 1000 rpm for 10s, followed by 3000 rpm for 45 s in a glove box at the relative humidity below 20%. Similarly, thin films of ASD DLM free base were prepared from a solution of DLM and HPMCP in MeOH-DCM 1:1 v/v. ASD films were stored under a vacuum oven overnight. Acidic media (HCl solution pH 1.6; or phosphate buffer pH 3.0, or pH 5.0) was added to a slide with a concave depression (Fisher Scientific, Pittsburgh, PA). The cover glass was placed in contact with the aqueous media. Drug crystallization on the film was visualized under PLM for 60 min using a 20× objective.

### 2.7 Scanning Electron Microscopy

Morphology of ASD particles and ASD films (prepared by the spin-coating method as described above) before and after incubation in acidic media was examined by a Nova nanoSEM field emission scanning electron microscope (FEI Company, Hillsboro, OR). Samples were mounted on an aluminum stub using double-sided sticky carbon tape and coated with a thin film of platinum using a sputter coater (Cressington Sputter Coater, Watford, UK) for 60 s. SEM images were obtained using an Everhart–Thornley detector with a spot size of 3 nm, beam energy of 5 kV, and working distance of approximately 5 mm.

## 3 Results

### 3.1 Solubility of DLM and Polymers as a Function of pH

**Figure 1:**
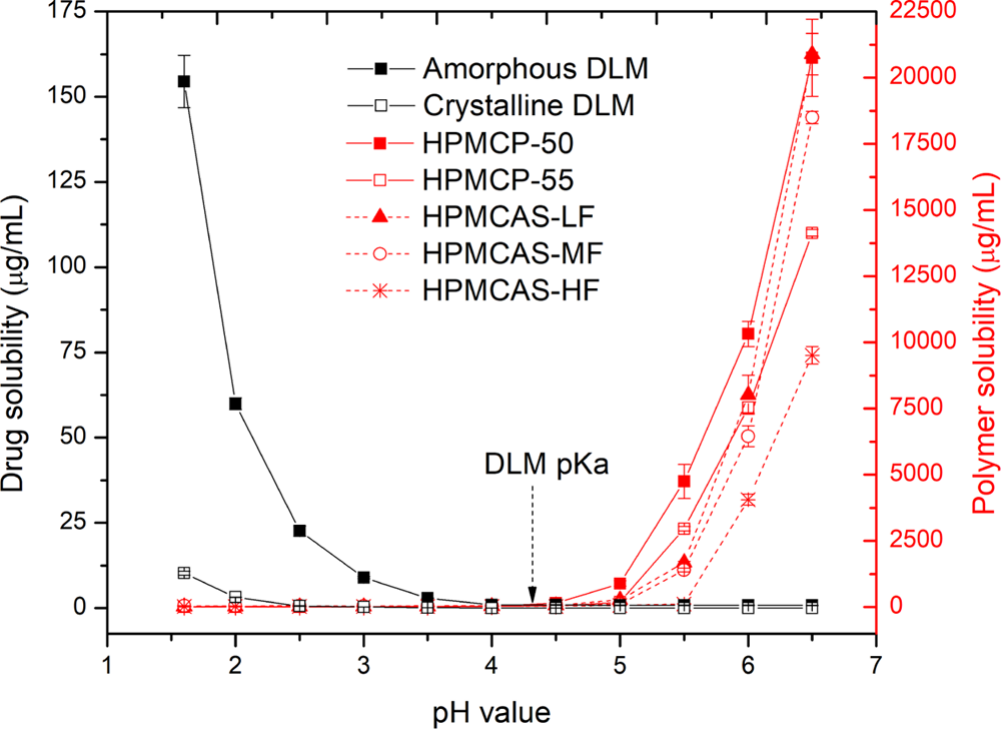
Solubility of drug and polymers (measured by HPLC-ELSD) in various pH media. The reported p*K*_a_ of DLM^49^ is noted by the arrow.

The solubility profiles for DLM and the various polymers over the pH range 1.6–6.5 are summarized in Figure 1. DLM is a weak base with a reported p*K*_a_ of 4.3.^49^ At pH values close to or higher than the drug p*K*_a_, the solubility was very low. At pH 6.5 where DLM is unionized, the crystalline solubility was 0.018 ± 0.003 μg/mL, while the amorphous solubility was 0.76 ± 0.06 μg/mL. Thus, the supersaturation ratio (SR) at the amorphous solubility (i.e., amorphous solubility/crystalline solubility) was 42. As the pH decreased below the p*K*_a_, to values found in the fasted gastric environment, both crystalline and amorphous solubility values increased. At pH 1.6, the crystalline and amorphous solubilities were 10.2 ± 0.6 and 154.5 ± 7.7 μg/mL, respectively. Thus, an increase in pH from 1.6 to 6.5 resulted in an approximately 500-fold decrease in crystalline solubility. The calculated p*K*_a_ of DLM according to the Henderson–Hasselbalch equation for weak bases was 3.94 ± 0.04 based on fitting the experimental solubility data (Figure S1). The solubility profiles enabled calculation of the extent of supersaturation achieved during the release studies.

In contrast to DLM, the enteric polymers were insoluble in media with a pH below 4.5, consistent with expectations and in line with their chemical properties (Table 3). Similar values for polymer solubility were obtained for the two analytic methods (colorimetric and HPLC-ELSD). HPMCP is a cellulose derivative with three types of substituents, including phthalyl, methoxy, and hydroxypropoxyl, with a mass content of phthalyl group of 21–35 wt %.^50^ HPMCP-50 has a p*K*_a_ of about 4.2.^13,51,52^ The solubility of HPMCP-50 at pH 5.0 was 893 ± 95 μg/mL. HPMCP-55 has been reported to have a higher p*K*_a_ (4.49^51^ and 4.83^53^) and the measured concentration at pH 5.0 was 189 ± 18 μg/mL, notably lower than that observed for HPMCP-50.

**Table 3:** p*K*_a_ and Dissolution pH Threshold of HPMCAS and HPMCP Polymers

polymer	p*K*_a_	dissolution pH threshold
HPMCP-50	4.20;^51^ 4.32^13^	5.0^55^
HPMCP-55	4.49;^51^ 4.83^53^	5.5^55^
HPMCAS-LF	5.09^53^	5.5;^54^ 4.8^50^
HPMCAS-MF	4.92;^13^ 5.0^56^	6.0;^54^ 5.2^50^
HPMCAS-HF	5.15^53^	6.8;^54^ 5.7^50^

For HPMCAS, the succinoyl group (mass content of 4–28 wt %) has a p*K*_a_ of about 5.0,^50,54^ consistent with the low solubility of the polymers below pH 5.0. At pH 5.5, the solubility of HPMCAS-LF and HPMCAS-MF increased steeply (1687 ± 47 and 1401 ± 69 μg/mL, respectively), where the -LF grade was more soluble due to a higher succinoyl content. Owing to the higher ratio of acetyl to succinoyl groups, the solubility of HPMCAS-HF was much lower than that of HPMCAS-LF or -MF grades and this polymer only dissolved at a pH of about 6.0 or higher. At pH 6.5, HPMCP-50 and HPMCAS-LF showed the highest solubilities, >20 mg/mL. The polymer solubility at pH 6.5 followed the order of HPMCP-50 ≈ HPMCAS-LF > HPMCAS-MF > HPMCP-55 > HPMCAS-HF.

Considering the experimental pH values used for in vitro release studies, the solubility of DLM and the polymers can be summarized as follows: at pH 1.6, DLM had appreciable solubility, in particular the amorphous form, whereas the polymers were insoluble. At pH 3.0 and 5.0, both drug and polymer exhibited low solubility, whereas at pH 6.5, the polymers were highly soluble, while the drug showed an insignificant solubility.

### 3.2 Release Profile and Drug Crystallization Behavior of DLM Free Base ASDs

DLM free base has a high tendency to crystallize during the manufacturing process and storage. The rapid crystallization of the drug was revealed by the fact that it was not possible to prepare an amorphous ASD with the free base at a 25% drug loading by rotary evaporation. However, using spray drying, an initially amorphous formulation could be successfully prepared (Figure S2A,B).

**Figure 2:**
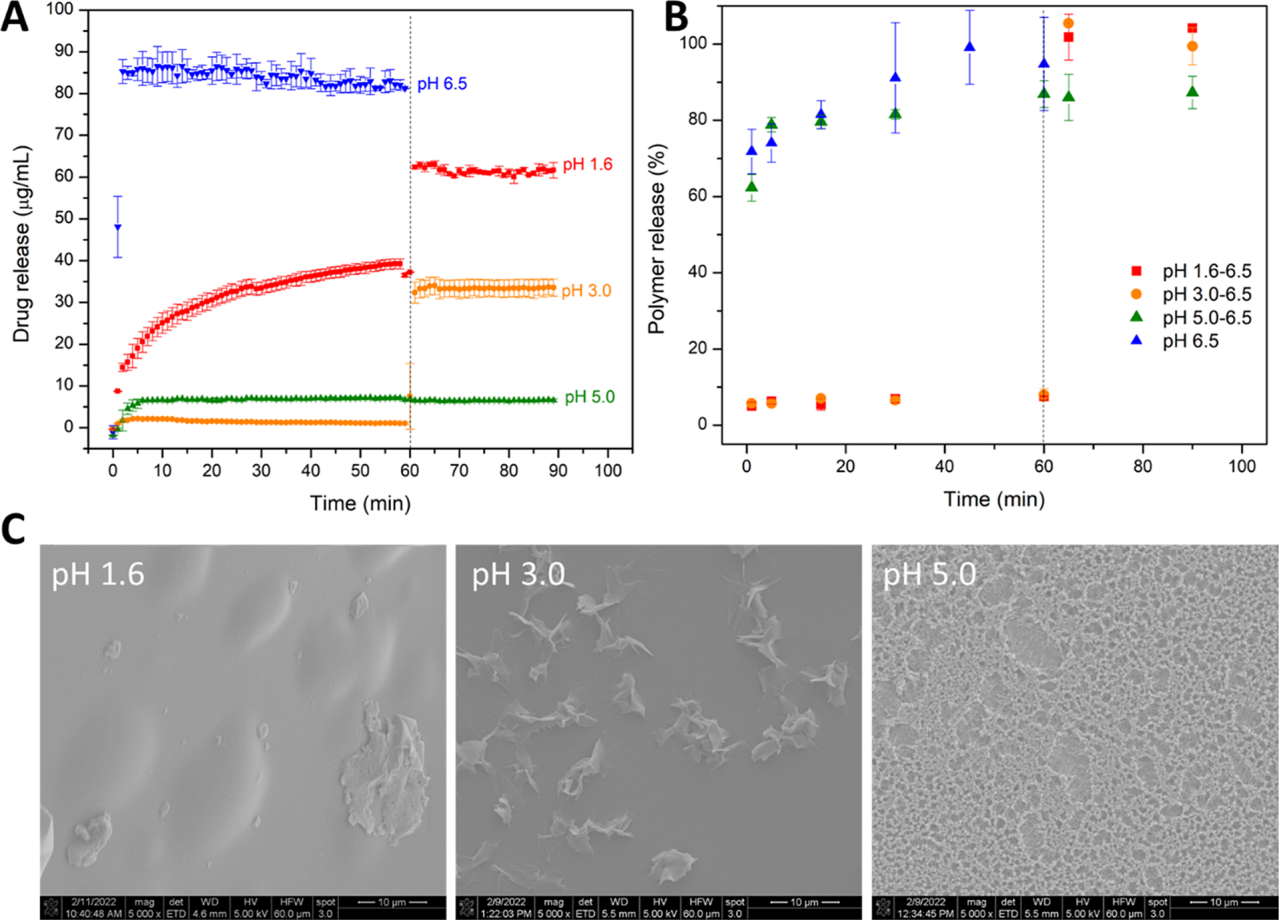
Release profile of DLM-HPMCP-50 ASD (at 25% drug loading, formulated as a disintegrating tablet). (A) Drug release profile; (B) polymer release profile. Dashed lines indicate pH shift from acidic media (pH 1.6; 3.0; and 5.0) to the intestinal fluid (pH 6.5). The final concentration for complete drug release in intestinal fluids is 100 μg/mL. (C) Crystallization behavior of ASD film after 1 h immersion in acidic media confirmed by SEM images.

The drug and polymer release profiles from tablets were evaluated in single-stage dissolution (phosphate buffer pH 6.5) and pH-shift experiments for ASDs with a drug loading of 25%. The spray-dried ASD of DLM base exhibited rapid and near complete release of both drug (>80%) and polymer at pH 6.5 (Figure 2A,B). In our previous study with the corresponding ASD of the free base prepared by rotary evaporation, the release extent was only 20%.^15^ This serves as confirmation that the spray-dried form was predominantly amorphous (consistent with PXRD data, Figure S2A), in contrast to the rotary-evaporated ASD, which was found to contain residual crystallinity. Drug release from ASDs containing DLM base varied depending on the pH of the gastric immersion stage. The total release following pH shift was greater for pH 1.6 versus pH 3.0 or 5.0. The lack of release was not due to poor polymer release; polymer released when the pH was increased for all systems (Figure 2B) and did not trend with drug release (Figure 2A). SEM results (Figure 2C) and PLM images (Figure S2C) indicated that crystallization occurred in all simulated gastric fluids.

### 3.3 Release Profiles and Drug Crystallization Behavior of DLM Edisylate ASDs

#### 3.3.1 Drug and Polymer Release Profile as a Function of pH

**Figure 3:**
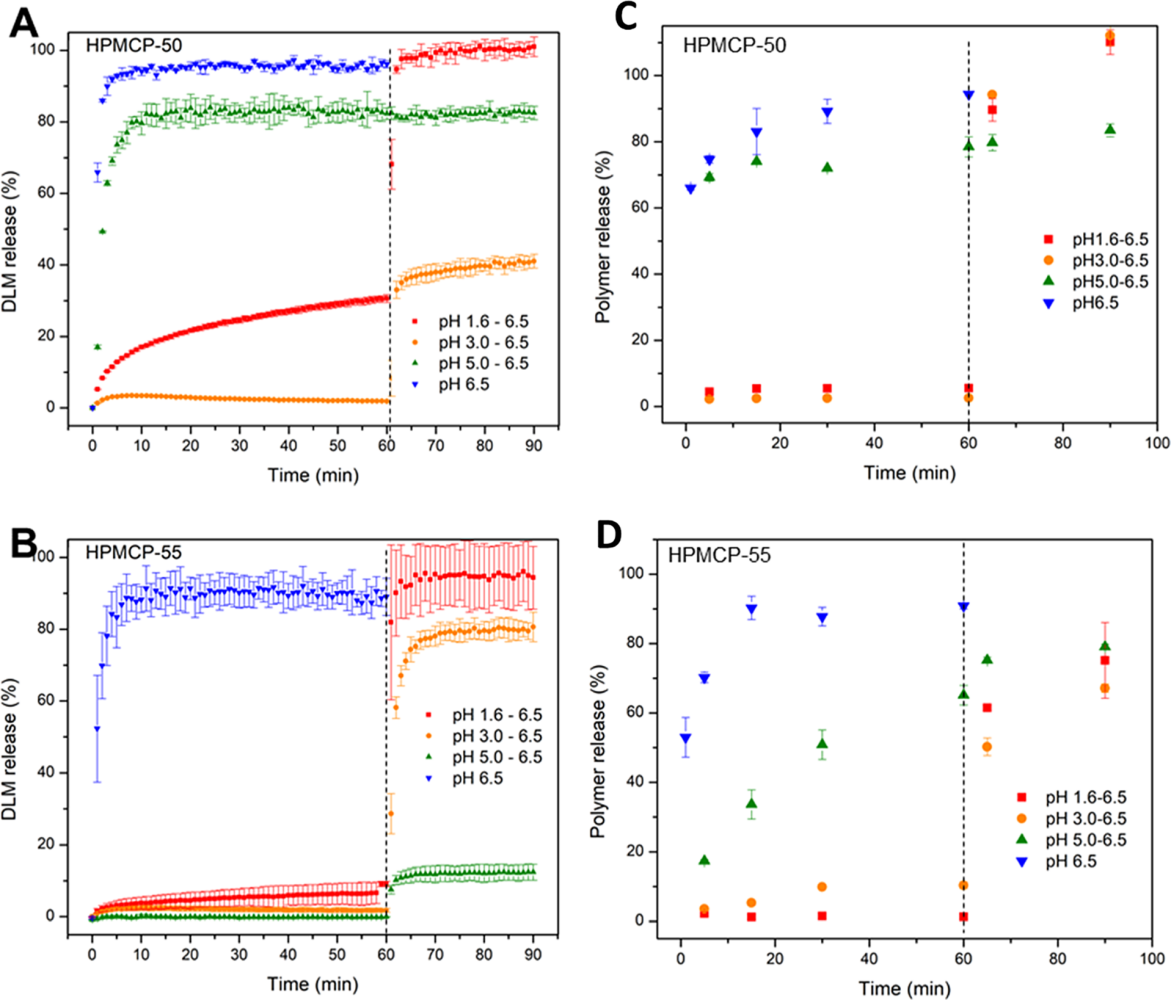
Drug (A,B) and polymer (C,D) release profiles of DLM edisylate ASDs (25% drug loading ASD, formulated as a disintegrating tablet) with (A,C) HPMCP-50 and (B,D) HPMCP-55. Dashed lines indicate pH shift.

It was of interest to evaluate if salt formation rendered drug release more robust to variable gastric immersion pH conditions. Similar to the free base ASDs, DLM edisylate ASDs with HPMCP-50 also showed rapid and near complete drug release at intestinal pH conditions (Figure 3A). Moreover, salt formation resulted in enhanced drug release in pH shift experiments for gastric pH conditions of 1.6 or 5.0. However, for an intermediate gastric stage pH value of 3.0, much lower release was also observed for DLM edisylate ASDs, indicating that salt formation only partially remediated variation in release extent with gastric stage pH. The release profiles of HPMCP-50 from ASDs containing the DLM edisylate salt (Figure 3C) were similar to those observed for the DLM free base ASDs (Figure 2B).

**Figure 4:**
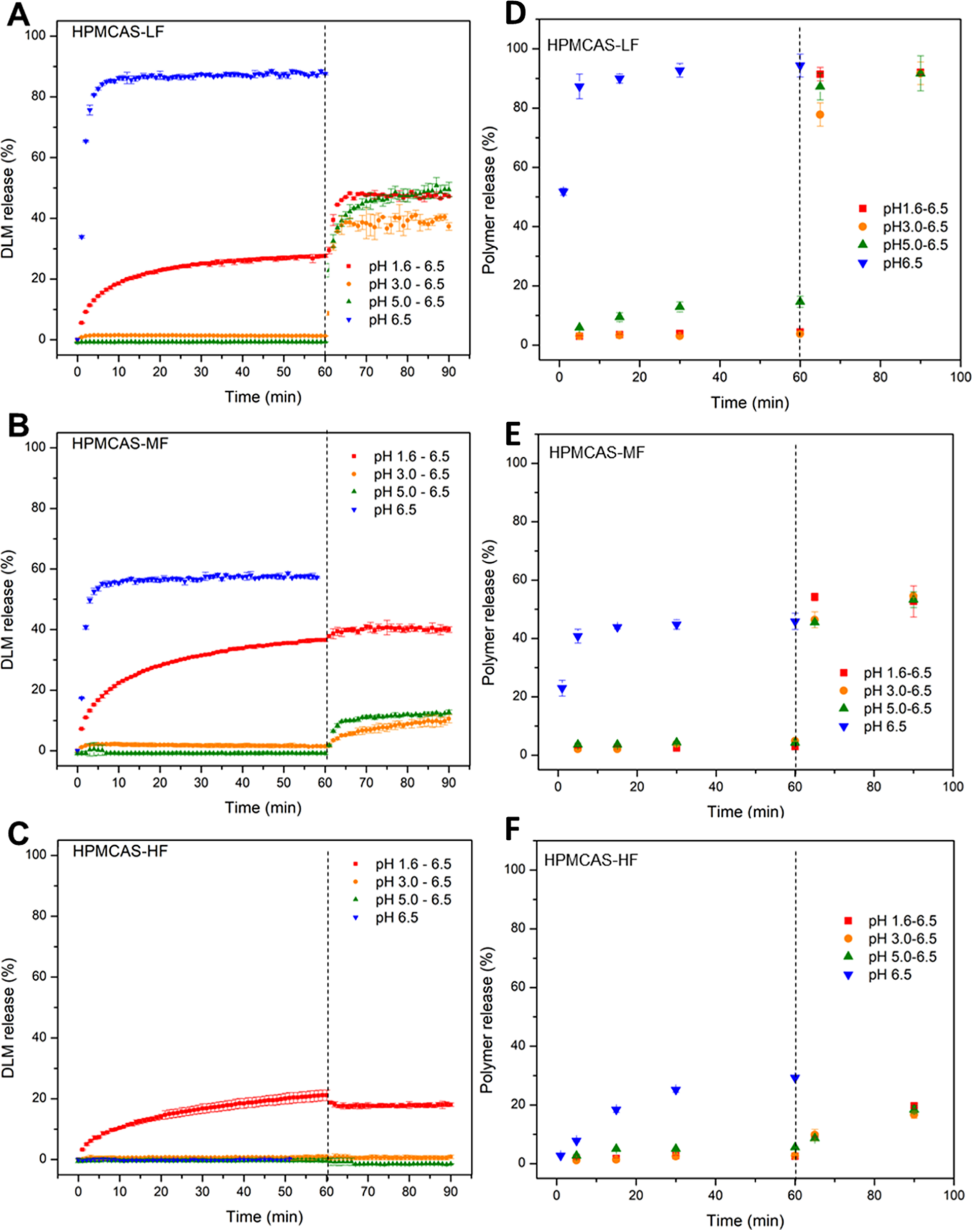
Drug (A–C) and polymer (D−F) release from ASDs of DLM edisylate (25% drug loading ASD, formulated as a disintegrating tablet) with (A,D) HPMCAS-LF; (B,E) HPMCAS-MF; and (C,F) HPMCAS-HF. Dashed lines indicate pH shift.

The impacts of different polymer types and grades on the release profiles of DLM edisylate salt ASDs were further investigated, with results shown in Figure 3 and Figure 4, respectively. Similar to HPMCP-50, good release profiles for both drug and polymer were observed at pH 6.5 for DLM edisylate ASDs with HPMCP-P55 (Figure 3B,D). In pH shift experiments, the overall release extent of the drug as well as the release profile varied, being dependent on the pH of the initial gastric stage, as well as the polymer grade. At pH 1.6, drug release in the gastric stage from ASDs with HPMCP-50 was higher than for HPMCP-55 (30% and <10% for HPMCP-50 and HPMCP-55, respectively). Near complete release was subsequently observed from both ASDs when the dissolution medium was shifted to pH 6.5. Drug release from these ASDs was very different with gastric stages of pH 3.0 and pH 5.0. At pH 3.0, little drug released in the gastric stage. Upon switching to intestinal conditions, HPMCP-55 ASDs led to ∼80% release, while only about 40% drug release was observed for HPMCP-50 ASDs. On the other hand, ASDs with HPMCP-55 exhibited no drug release at pH 5.0, followed by a very low release at pH 6.5, while the corresponding ASD with HPMCP-50 had good release at pH 5.0, but no additional release upon transfer to pH 6.5 media. In terms of polymer release, HPMCP-50 and P-55 shared similar release profiles at pH 1.6 and 3.0, with minimal release in acidic media, followed by rapid release at pH 6.5, where the release of HPMCP-50 at higher pH was faster than that of HPMCP-55. Similarly, both polymers could dissolve at pH 5.0, but the release rate of HPMCP-55 was much slower than that of HPMCP-50.

For ASDs of DLM edisylate and various grades of HPMCAS, near complete release was observed only in phosphate buffer pH 6.5 and for the -LF grade (Figure 4A). Under corresponding conditions, drug release from the -MF grade was about 55% (Figure 4B), while there was almost no release of DLM from the ASD prepared with HPMCAS-HF (Figure 4C). The polymer release profile was quite consistent with the drug release profile where the extent of polymer release followed the order HPMCAS-LF > HPMCAS-MF > HPMCAS-HF (Figure 4D–F). In pH shift experiments, all ASD formulations had a similar pattern of drug release, with the greatest extent of release in the gastric compartment occurring in lower pH media. However, when the pH of the solution was shifted to 6.5, none of ASDs exhibited near complete drug release with the highest extent of dissolution observed for HPMCAS-LF ASDs. Due to the poor solubility of HPMCAS at low pH, there was very low polymer release in an acidic environment. The extent of polymer release after transition to pH 6.5 followed the order of HPMCAS-LF > -MF > -HF.

#### 3.3.2 Drug Crystallization during Incubation in Acidic Environments

**Figure 5:**
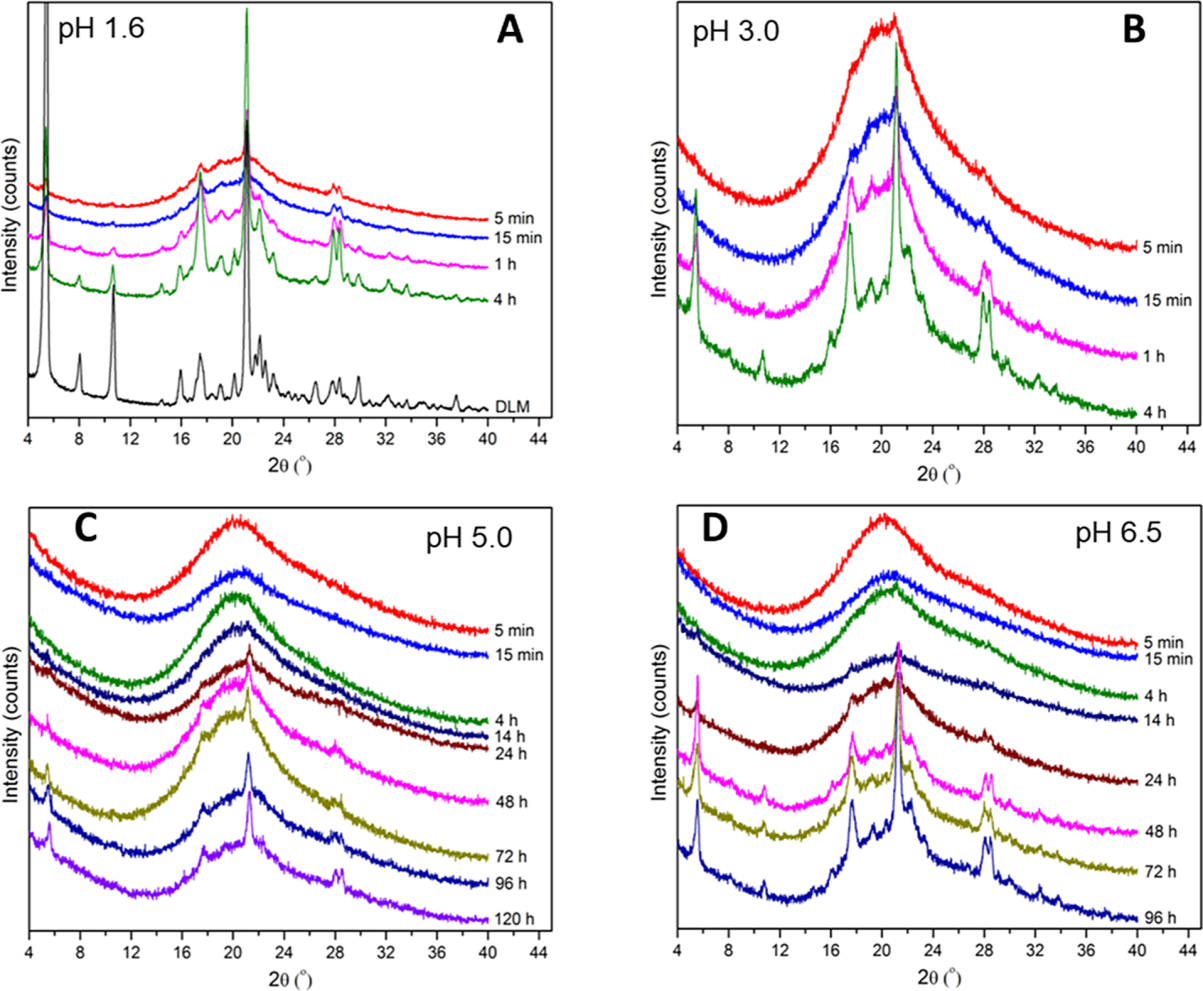
Crystallization of neat amorphous DLM edisylate as a function of incubation time in media of different pH values. (A) pH 1.6; (B) pH 3.0; (C) pH 5.0; and (D) pH 6.5.

PXRD and SEM were utilized to evaluate drug crystallization after immersion of neat amorphous salt and various ASDs in different pH media. For the neat edisylate salt, it is apparent from Figure 5A,B that crystallization was rapid at both pH 1.6 and 3.0. Crystallization to the free base was observed, indicating that the pH_max_ of the system is lower than pH 1.6;^57^ in other words, the free base was the stable crystalline form for all of the pHs investigated herein, and hence the salts converted to this form. Consequently, the amorphous salt underwent disproportionation followed by crystallization to the free base. The rapid conversion to the crystalline free base (evidence of free base was seen after 5 min immersion) is consistent with previous studies on the neat salt, where only a small extent of supersaturation was seen following dissolution at pH 1.6.^15^ However, the rate of crystallization was much reduced for the salt suspensions held at pH 5.0 and 6.5 (Figure 5C,D), likely due to a reduced driving force for dissolution at this pH due to a lower solubility than at pH 1.6 or pH 3.0.

**Figure 6:**
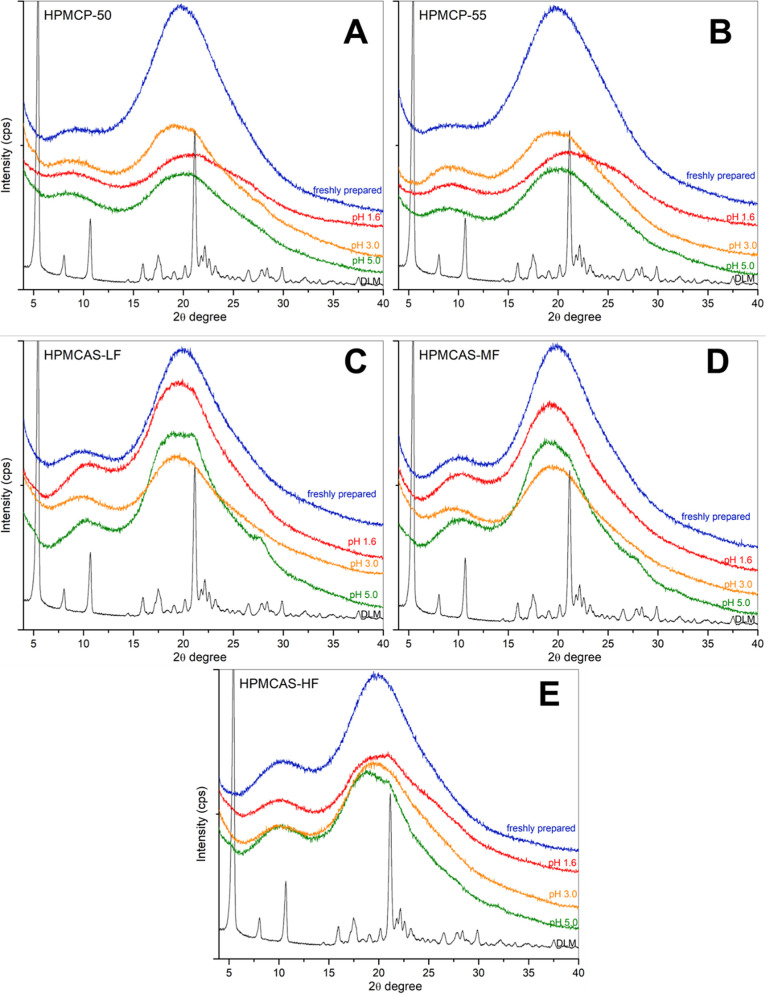
Diffractograms of DLM edisylate ASD powder with (A,B) HPMCP and (C–E) HPMCAS at 25% drug loading after incubation in an acidic environment for 1 h.

**Figure 7:**
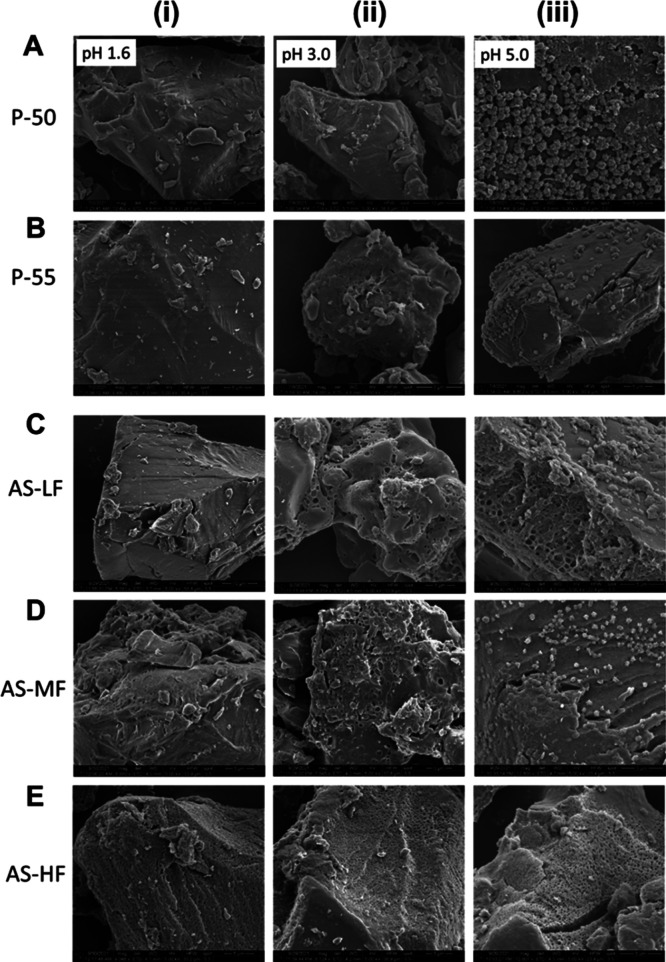
SEM images of DLM edisylate ASD powders with (A,B) HPMCP and (C–E) HPMCAS at a 25% drug loading after incubation at pH 1.6 (i), pH 3.0 (ii), and pH 5.0 (iii) for 1 h.

Drug crystallization could not be detected via PXRD for DLM edisylate salt ASDs following suspension in acidic environments for up to 1 h (Figure 6). However, the appearance of crystals on the surface of ASD particles was evident from SEM images of ASD particles after incubation (Figure 7) versus before incubation (Figure S3). For HPMCP ASDs, no changes to the ASD particle surfaces were observed for either polymer grade for pH 1.6 media (Figure 7Ai,Bi). Moreover, crystals started appearing following suspension in intermediate pH media for HPMCP-50 ASDs, in particular for pH 5.0 media, where numerous small agglomerates were present at the surface (Figure 7Aii,iii). For HPMCP-55 ASD, crystallization was also noted at pH 5.0, albeit to a lower extent (Figure 7Biii). A higher crystallization tendency was observed for the HMPCAS ASDs (Figure 7C–E). The particle surfaces appeared porous, especially for HPMCAS-HF ASDs, and crystal agglomerates were present (Figure 7E). ASDs with the three HPMCAS grades shared similar patterns of increased drug crystallization as the solution pH increased. Furthermore, after incubation in acidic pH, followed by pH shift to pH 6.5, any undissolved particles rapidly became covered in small crystals after 30 min in the higher pH solution (Figure S4).

**Figure 8:**
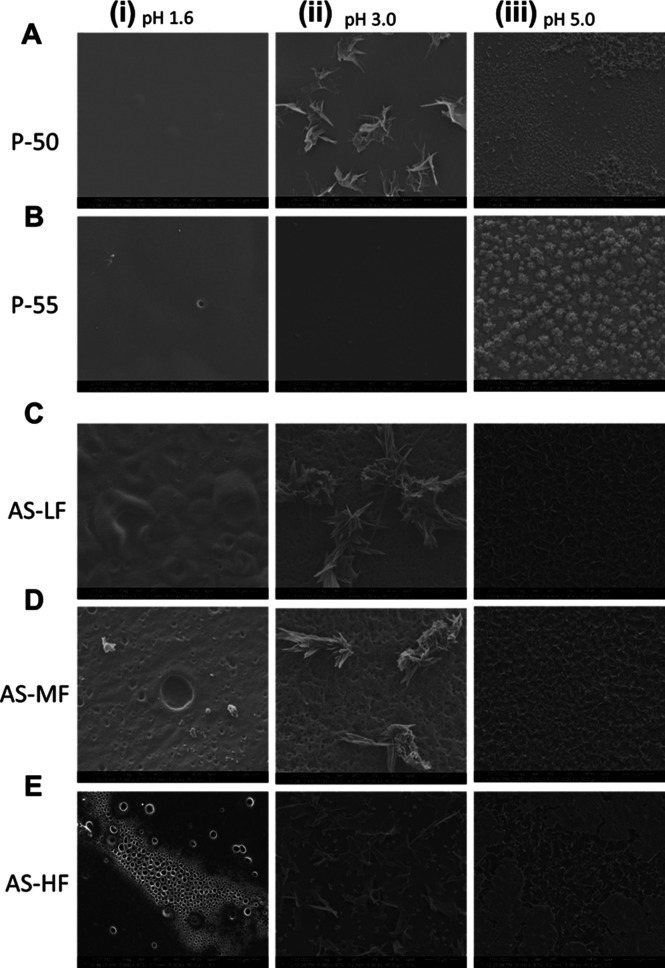
SEM images of DLM edisylate ASD films with (A,B) HPMCP and (C–E) HPMCAS after incubation in pH 1.6 (i), pH 3.0 (ii), or pH 5.0 (iii) for 1 h.

To further investigate the crystallization tendency of the various ASDs in an acidic environment, incubation of ASD films in different media was performed. In agreement with SEM images of HPMCP ASD powders, there were no notable visual changes to the ASD film after 1 h incubation in pH 1.6 HCl solution, with PLM images shown in Figure S5A and SEM images in Figure 8Ai,Bi. In contrast, crystallization was observed for HPMCAS-HF grade (Figure S5B and 8Ei). At pH 3.0, all ASDs showed evidence of crystallinity (Figure 8ii) with the exception of the DLM edisylate-HPMCP-55 formulation (Figure 8Bii). For pH 5.0, the surface of the ASD films showed high coverage of crystals that were smaller than those observed at pH 3.0 (Figure 8iii). Partial dissolution of the DLM edisylate-HPMCP-50 ASD was observed; however, crystallization was detected on undissolved regions of the film (Figure 8Aiii). In general, the tendency of the drug to crystallize in an acidic environment was higher for ASDs with HPMCAS than with HPMCP.

## 4 Discussion

Following oral administration, a dosage form first encounters the stomach, which has a highly variable pH that depends on the prandial state and intra- and interindividual variations. Gastric pH is low in the fasted state and increases notably in the presence of food.^40,41,58,59^ Depending on the characteristics of the dosage form, partial dissolution can occur in the gastric compartment, but little-to-no absorption is expected due to the low surface area of the gastric mucosa. The gastric residence time also depends on the prandial state, with values typically in the range of 15–60 min for the fasted state^60,61^ and 2–5 h for the fed state.^40−42,58^ Historically, dissolution medium pH is an important variable, known to impact release from many dosage forms, in particular those containing ionizable APIs^26^ or an enteric coating.^42,62,63^ In addition, buffer capacity, ionic strength, and buffer type have been observed to impact drug release^64−66^ as well as polymer dissolution.^52^ For example, Qi and Taylor reported that the release rate of HPMCP-50 in 50 mM pH 6.8 buffer varied depending on cationic species and buffer type.^52^ The dissolution rate of 2-naphthoic acid was found to improve at higher pH and higher buffer capacity, but the extent of increase depended on buffer type.^66^ Similarly, the release rate of nifedipine from ASDs with HPMCAS in bicarbonate buffer was much slower than in phosphate buffer as reported by Sakamoto and Sugano.^65^ Meanwhile, an increase of ionic strength decreased the release of diltiazem hydrochloride from HPMC matrices.^67^ For metastable formulations, such as ASDs, supersaturation may be generated following dissolution. Thus, for these systems, it is essential to study not only release kinetics, but also any underlying phase transitions that occur simultaneously since these can change the trajectory of subsequent dissolution processes. For ASDs containing an API and/or polymer with pH-dependent solubility, consideration of the impacts of different gastric environments on release, supersaturation, and phase behavior emerges as a complex paradigm requiring study.

DLM has very low aqueous solubility when the pH is above the pK_a_ but shows appreciable solubility at pH 1.6 (Figure 1), in particular when in the amorphous form. However, the polymers used to form the ASD are poorly soluble at low pH. This creates an interesting scenario around whether enteric polymers, when used as the matrix in an ASD formulation, can prevent drug release under fasted state gastric conditions, lower the extent of supersaturation (if any) that develops during immersion in the gastric compartment, and reduce the risk for crystallization during the gastric retention period. Indeed, despite the low polymer solubility at pH 1.6, a notable amount of drug was released at this pH for all ASDs (Figures 3 and 4). The observation of drug release from enteric polymer ASDs at low pH has been reported for other weakly basic compounds including posaconazole,^27^ clotrimazole,^26^ ketoconazole,^68^ and itraconazole.^69^ For posaconazole, maximum concentrations achieved by ASDs with HPMCAS 10–50% DL in FaSSGF were below the crystalline solubility at that pH.^27^ However, high DL ASDs exhibited more drug leaching in gastric conditions and achieved supersaturation.^70^ Similarly, for clotrimazole-HPMCAS ASDs, supersaturated solutions were generated, although the lowest pH studied was pH 3.^26^

**Figure 9:**
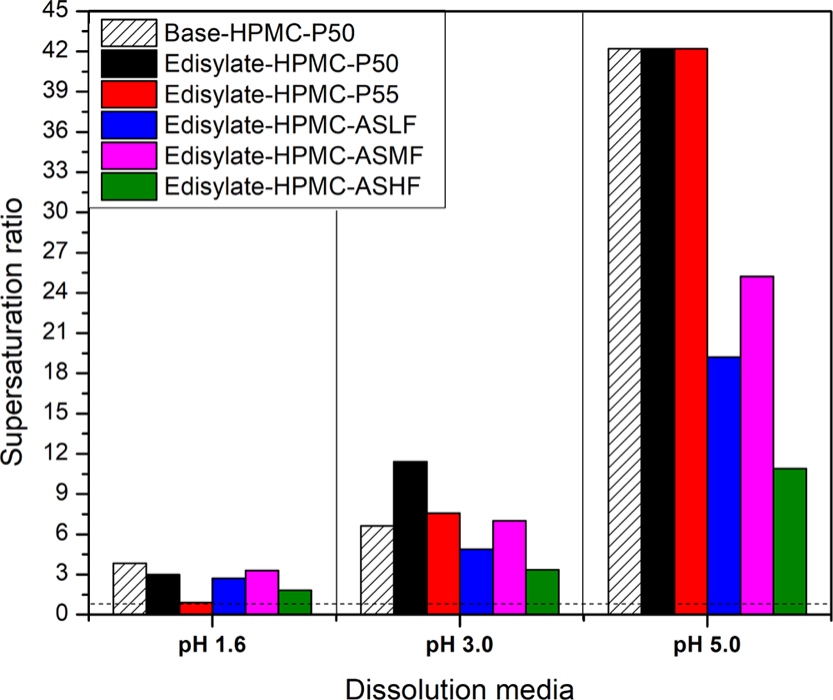
Maximum supersaturation ratio following immersion of ASDs in acidic dissolution media for 1 h.

In the case of DLM ASDs, the extent of drug release and supersaturation observed after 60 min depended on the medium pH. For most ASDs, the concentration after 60 min of immersion exceeded the crystalline solubility and therefore a supersaturated solution was generated in the gastric compartment (Figure 9, when the SR is >1, the solution is supersaturated), although the extent of supersaturation varied depending on the pH. The low extent of supersaturation at pH 1.6 can be attributed to the higher drug solubility at this pH; hence even though the released concentrations were higher, this translated into a lower extent of supersaturation. This follows because the SR is approximated as the solution concentration/crystal solubility, and the crystalline solubility is increased at pH 1.6. The highest supersaturation was noted at pH 5.0 where some of the ASDs released sufficient DLM to reach the supersaturation limit dictated by the drug amorphous solubility. Thus, the general trend for all of the ASDs, regardless of the polymer, was that the SR in the gastric compartment increased as the pH was raised from 1.6 to 5.0. The extent of release into the medium is an important consideration when evaluating ASD failure mechanisms because supersaturation provides the driving force for crystallization, both in the gastric compartment and following transfer to intestinal pH conditions.

Notably, over the residence period, there was little evidence of desupersaturation via crystallization from the solution phase in the gastric compartment, since no depletion in the achieved concentration with time was generally observed. To verify that solution crystallization was not the main failure route, release studies were performed on selected ASDs in the presence of predissolved PVPVA, which has previously been found to be a good inhibitor of solution crystallization for DLM.^15^ The presence of PVPVA did not affect the drug release of DLM edisylate ASDs with HPMCAS-MF and HPMCP-50, as seen in Figure S6. The lack of crystallization from the solution phase may be due to the presence of a minor amount of dissolved polymer at all pH conditions (Figure 1). Previous studies have shown that cellulose derivatives are highly effective inhibitors of DLM solution crystallization, even at high levels of supersaturation.^15^ At pH 5.0 where the thermodynamic driving force for crystallization was highest (high SR), polymer solubility exceeded 100 μg/mL in all instances with the exception of HPMCAS-HF (Figure 1). Further evidence that crystallization from the solution phase in the gastric compartment was not the predominant failure mechanism was provided by the observation that there was no link between the extent of supersaturation in the gastric compartment and the eventual release level upon transfer to intestinal pH conditions. This is readily apparent by considering the extent of supersaturation observed at pH 3.0 for the different ASDs, where there were relatively minor differences, while subsequent release in the intestinal compartment was relatively poor for all ASDs with the exception of the HPMCP-55 system.

Given the observed extensive drug release with most ASDs immersed at pH 1.6, it is interesting to note that HPMCP-55 prevented appreciable drug release. Variations in polymer solubility do not account for the observed differences (Figure 1), whereby there was negligible polymer release for all systems at this pH (Figures 2 and 3). For ASDs where the polymer is essentially insoluble, the extent of drug release is expected to depend on the chemical potential of the drug in the ASD, which in turn is dependent on the drug loading, the ASD water content, as well as the nature and extent of any drug–polymer interactions in the ASD matrix.^6,26,71^ Poorly miscible systems where drug–polymer interactions are weak are expected to show the highest extent of drug release since the drug will have a higher chemical potential. Conversely, systems with strong/extensive drug–polymer interactions will show a lower extent of release.^26^ Given the low extent of release compared to ASDs with other polymers, it is inferred that HPMCP-55, which has a higher phthalyl content, higher molecular weight, and higher acid resistance than HPMCP-50,^55^ may form relatively stronger/more extensive interactions with the drug, leading to reduced release at a pH where the polymer is insoluble.

Besides solution crystallization, a second possible crystallization route was matrix crystallization. This occurred in the undissolved ASD matrix, which persisted in the gastric compartment due to the low solubility of the polymers below pH 5.0 (Figure 1). When ASD particles are immersed in solution, water will be absorbed, lowering the glass transition temperature and increasing the molecular mobility. This can lead to crystallization of susceptible drugs. For example, posaconazole ADS particles were found to undergo matrix crystallization when suspended in acidic medium, whereby the extent of crystallization increased with drug loading.^27^ Matrix crystallization impacts ASD performance because any crystallized material is less soluble and will have a lower tendency to dissolve. Further, crystals formed by matrix crystallization following immersion in the gastric compartment can act as seeds for additional crystal growth, since the seeds will be released into solution upon transfer to a higher pH environment where the polymer becomes soluble.^72^ In the case of DLM, the extent of matrix crystallization was very low, could not be detected by PXRD, where crystals likely formed predominantly at the surface; qualitative evidence of crystallinity was provided by PLM and SEM. Thus, matrix crystallization seems to be an important failure mechanism that occurs during suspension of the ASD in aqueous media, with the extent of surface crystallization varying with pH, polymer, and if the salt or free base form of the drug is present in the ASD. The mass fraction of crystals formed from ASDs during gastric immersion was low, given that crystallization was undetectable by PXRD (Figure 6), which is typically considered to have detection limits of about 1–5% crystallinity.^72−74^ Despite the low amount of crystals observed, they are potentially catastrophic to release performance, when the pH is increased to a value where the polymer becomes soluble.

**Figure 10:**
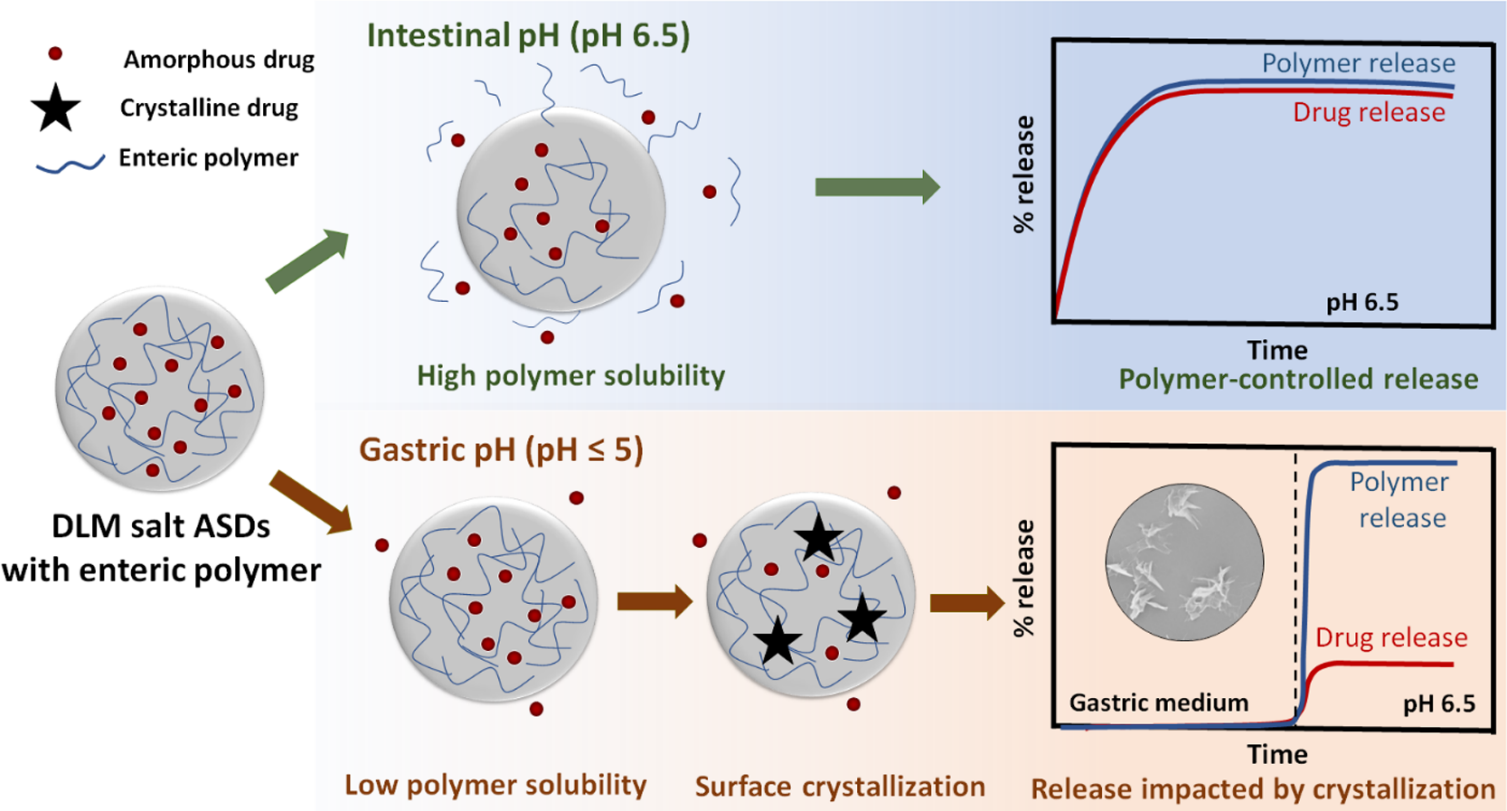
Schematic illustration of the drug release mechanism from DLM salt ASDs with an enteric polymer under different release conditions. The ASDs show near complete drug release at pH 6.5 where the polymer is highly soluble. At lower pH values, the polymer solubility is significantly reduced, facilitating surface drug crystallization, thereby hindering the drug release.

Due to the low mass of crystals formed, we were unable to investigate quantitative relationships between the extent of crystallization in the gastric compartment and release performance at pH 6.5. However, we can make note of some general trends which are illustrated in Figure 10. First, the presence or absence of surface crystallization, following gastric immersion at pH 1.6 (Figure 8i), largely correlates with the subsequent release performance upon transfer to a higher pH medium (Figures 3 and 4). Further, the incomplete release (only 20–65%, Figures 3 and 4) from ASDs exhibiting surface crystallization (Figure 8ii,iii) indicates that additional and rapid crystallization occurs after pH increase. Thus, it is likely that as the polymer started to dissolve when transferred to higher pH, a process that involves ionization and additional hydration and crystallization of amorphous drug in the vicinity of the crystal seeds occurred, although it should be noted that some additional drug release did take place upon transfer to a higher pH. Thus, there appear to be two competing processes, drug crystallization and drug release. Furthermore, it should be noted that once the drug entered the solution phase, the concentration level remained constant, indicating that the growth of the crystal seeds in the bulk solution was likely limited by the presence of the dissolved polymer (Figure S6). This is consistent with other studies that have demonstrated that polymers can adsorb to crystals and, depending on their surface coverage and conformation, block growth via step pinning.^24,75,76^

Second, formulating as a salt appears to have a positive impact in delaying crystallization when using HPMCP-50 as the ASD polymer. For example, no surface crystals were observed for the salt ASDs with HPMCP at pH 1.6 (Figure 8Ai), while the corresponding free base ASD was not physically stable under the same immersion conditions (Figures 2C and S2C). This was reflected in the lower release from the free base ASD relative to the comparable edisylate salt ASD, following immersion at low pH. The edisylate salt ASDs matched or outperformed release from the free base ASD at any given gastric compartment pH. Given that the crystals formed at all pH conditions were free base drug, it is likely that salt formation hindered crystallization by first requiring conversion to the free base, followed by crystallization. A previous study has shown that DLM salts had a lower tendency to crystallize relative to the free base form.^15^ Thus, salt formation provides a kinetic advantage resulting in slower crystal formation during acid immersion and consequently better release upon transfer to higher pH conditions.

Third, surface crystallization tendency varies with pH, where less crystallization was typically observed in solutions of lower pH relative to at pH 5.0, although this assessment was by necessity qualitative due to the experimental challenge of quantifying the extent of crystallization. Thus, release was typically (but not always) lower following immersion at pH 5.0 relative to pH 3.0, followed by transfer to pH 6.5, where crystallization visually appeared to be more extensive for particles harvested after pH 5.0 immersion (Figures 8 and S5). One outlier to the trend of more impaired release following immersion at pH 5, the edisylate salt HPMCP-50 ASD, can be explained by considering the polymer pH solubility profiles. This polymer showed a higher solubility than the other polymers at this pH, explaining the high extent of drug release observed in the pH 5.0 gastric compartment, which presumably mitigates the impact of any surface crystallization (in other words, there is a competition between drug matrix crystallization and drug release at this pH). Ideally, more sensitive methods to quantitate crystallinity in the different formulations would be available to provide greater insight into these apparent links between surface crystallization and subsequent release performance.

It is also apparent that DLM edisylate ASDs prepared with the family of HPMCAS polymers fared poorly during the two-stage dissolution experiments, with higher levels of surface crystallization, and low levels of eventual drug release. Part of their poor performance, at least for the MF and HF grades, can be attributed to the low extent of polymer release at pH 6.5 (Figure 4). This provides additional opportunity for matrix crystallization to occur at pH 6.5.

Taken in concert, the studies with DLM ASDs highlight the need to consider how variations in gastric pH that may arise in vivo due to factors such as inter- and intra-subject variability, age, prandial state, or coadministration of acid reducing agents impact the release performance of drugs from ASDs formulated with enteric polymers. In vitro dissolution testing is typically standardized in terms of media choice, and variations in gastric compartment pH are not commonly investigated for ASD formulations. However, for a weakly basic drug with a high tendency to undergo crystallization, gastric pH variations may be important in vivo in terms of how much drug is eventually absorbed. Further, given the sensitivity of the DLM formulations to gastric pH variability in terms of their release performance and the postulated impact on absorption, formulation strategies that mitigate drug crystallization risk at pH environments where the ASD polymer is insoluble clearly need to be explored.

## 5 Conclusions

Release performance of DLM ASDs formulated with enteric polymers, HPMCP or HPMCAS, was found to be impacted by the pH of the gastric compartment, the polymer type, and if the free base drug or an edisylate salt was used in the ASD. Surface crystallization following immersion in the gastric stage prior to transfer to intestinal conditions was demonstrated to be responsible for suboptimal drug release. Various grades of HPMCAS were found to be poor inhibitors of drug matrix crystallization at all pH conditions, while HPMCP grades were generally better inhibitors. The edisylate salt formulation allowed for improved release relative to the free base ASD, suggesting that salt formation offered some protection to gastric pH variations. Polymer solubility and polymer release also contributed to drug release behavior. In order to optimize the release profile of a drug that is sensitive to crystallization in conditions mimicking the gastrointestinal environment, it may be important to minimize contact of the drug with low pH conditions, such as by applying an enteric coating.

## Supporting Information Available

HPLC-ELSD conditions for polymer analysis; calculated p*K*_a_ based on fitting the experimental solubility data; characterization of ASD of DLM free base prepared by spray drying; morphology of freshly prepared DLM edisylate ASD particles; crystallization of ASD particles after pH-shift experiments; crystallization of DLM edisylate ASD films after incubation in acidic media; and drug release in media containing a predissolved polymer (PDF)

## Abbreviations

ASD: amorphous solid dispersion
BCS: biopharmaceutics classification system
DCM: dichloromethane
DLM: delamanid
DMSO: dimethyl sulfoxide
ELSD: evaporative light scattering
HPLC: high performance liquid chromatography
HPMCAS: hydroxypropyl methylcellulose acetate succinate
HPMCP: hydroxypropyl methylcellulose phthalate
MeOH: methanol
PLM: polarized light microscopy
PXRD: powder X-ray diffraction
SEM: scanning electron microscopy
SR: supersaturation ratio
UV: ultraviolet
