# Supplementary material for: Impact of Gastric pH Variations on the Release of Amorphous Solid Dispersion Formulations Containing a Weakly Basic Drug and Enteric Polymers
Source: Mol Pharm. 2023 Feb 2;20(3):1681–95. doi: 10.1021/acs.molpharmaceut.2c00895 (PMC9997068; doi:10.1021/acs.molpharmaceut.2c00895)
Supplement: Supplementary file 1 — mp2c00895_si_001.pdf [file mp2c00895_si_001.pdf]

SUPPLEMENTARY MATERIAL

Impact of gastric pH variations on the release of amorphous solid dispersion formulations containing a weakly basic drug and enteric polymers

Hanh Thuy Nguyen[1, §], Tu Van Duong[1, §], and Lynne S. Taylor[1, *]

[1] Department of Industrial and Physical Pharmacy, College of Pharmacy, Purdue University, West Lafayette, Indiana 47907, United States

[§]Authors contributed equally.

[*] Corresponding author. E-mail: lstaylor@purdue.edu. Tel: +1 (765) 496-6614. Fax: +1 (765) 494-6545.

Table S1: Chromatographic conditions for HPLC-ELSD to determine polymer concentration [1-2]

Mobile phase	Solvent A: 0.1% (v/v) formic acid in water		
	Solvent B: 0.1% (v/v) formic acid in acetonitrile		
Gradient	Time (min)	% Solvent A	% Solvent B
	0	70	30
	3	70	30
	3.01	65	35
	6	65	35
	6.01	10	90
	18	10	90
	18.01	70	30
	25	70	30
Flow rate	0.5 mL/min		
Injection volume	50 µL		
Column	Shodex RSpak DS-413 (3.5 µm, 4.6 mm × 150 mm)		
ELSD settings	Gas flow rate = 1.5 standard liter per minute;		
	Nebulizer temperature = 80 °C; evaporator temperature = 85 °C		
Run time	25 min		

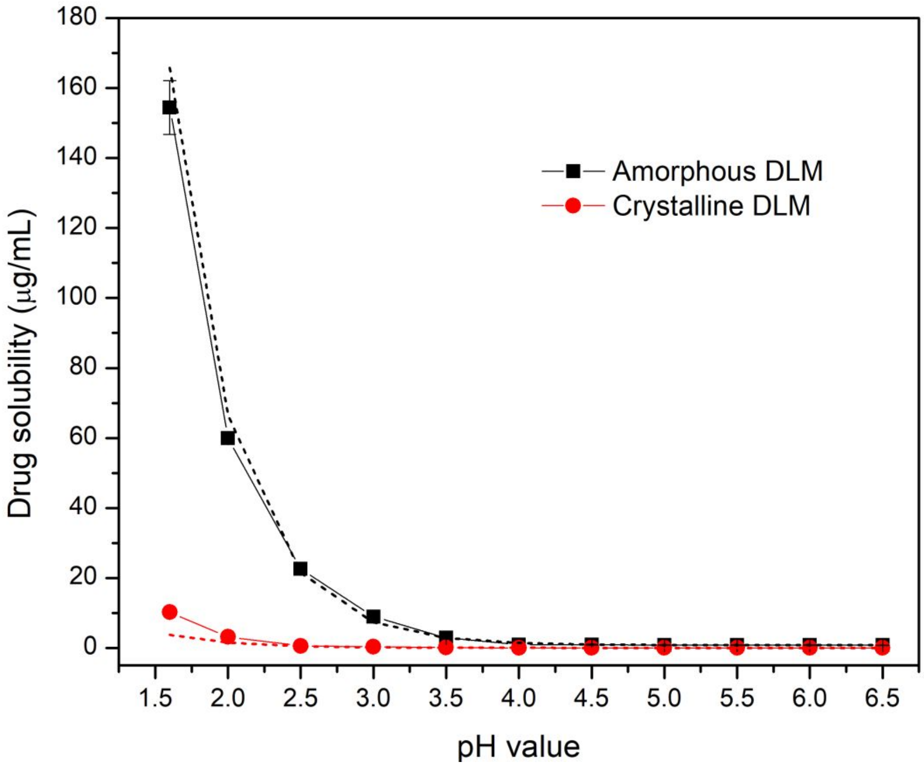

Figure S1: Solid lines indicate DLM solubility as a function of pH at 37 °C in phosphate buffer across the pH range 1.6–6.5. Dashed lines refer to the solubility values predicted by the Henderson-Hasselbalch equation using a pK_a value of 3.94.

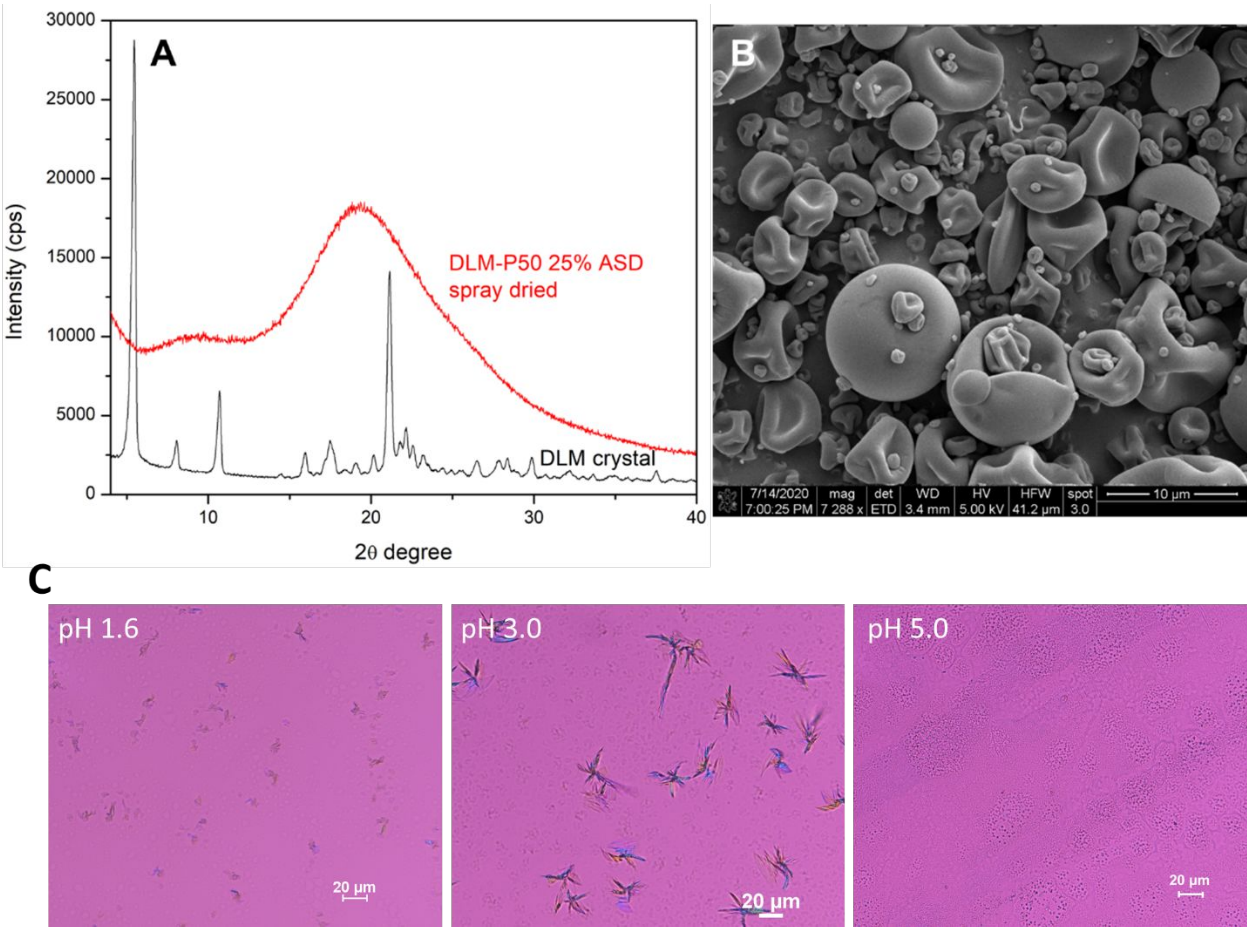

Figure S2: Predominantly amorphous state of freshly prepared spray dried DLM-HPMCP-50 ASD 25% DL confirmed by (A) PXRD and (B) SEM images. (C) Crystallization after acidic incubation for 1h as visualized by PLM

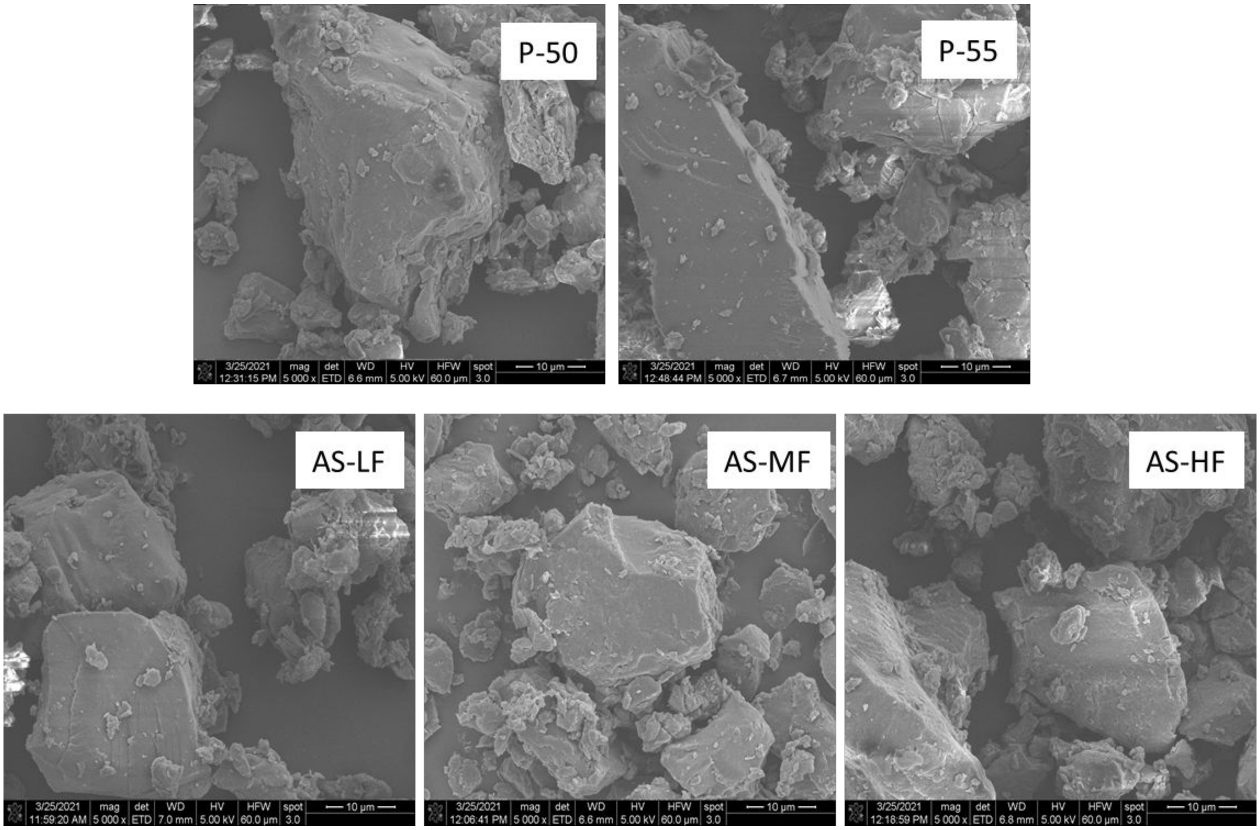

Figure S3: SEM images of freshly prepared DLM edisylate ASDs with HPMCP and HPMCAS of different grades at 25% DL.

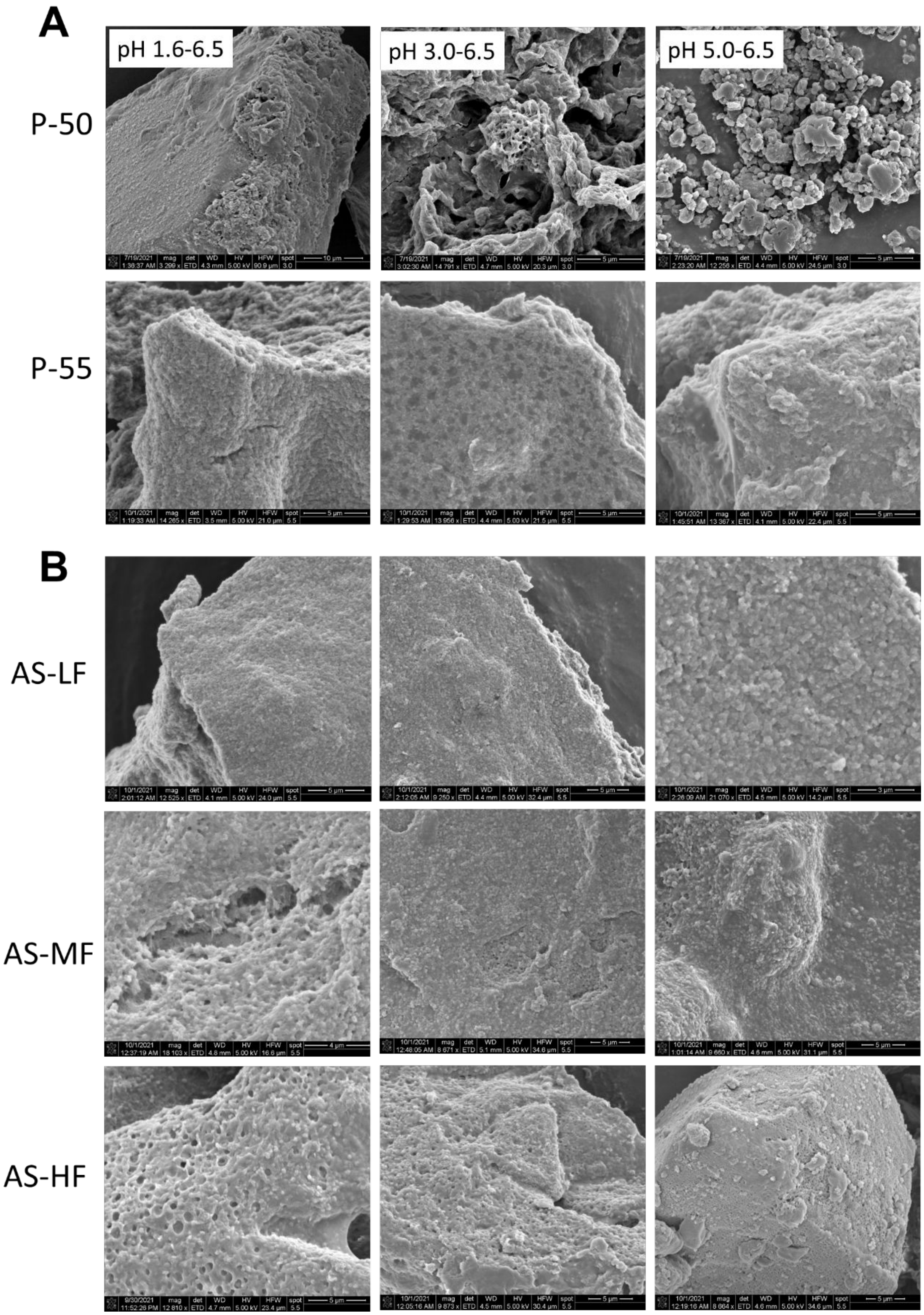

Figure S4: SEM images of DLM edisylate ASDs with HPMCP and HPMCAS at 25% DL after immersion in acidic solution for 1h following by 30 min in PBS pH 6.5

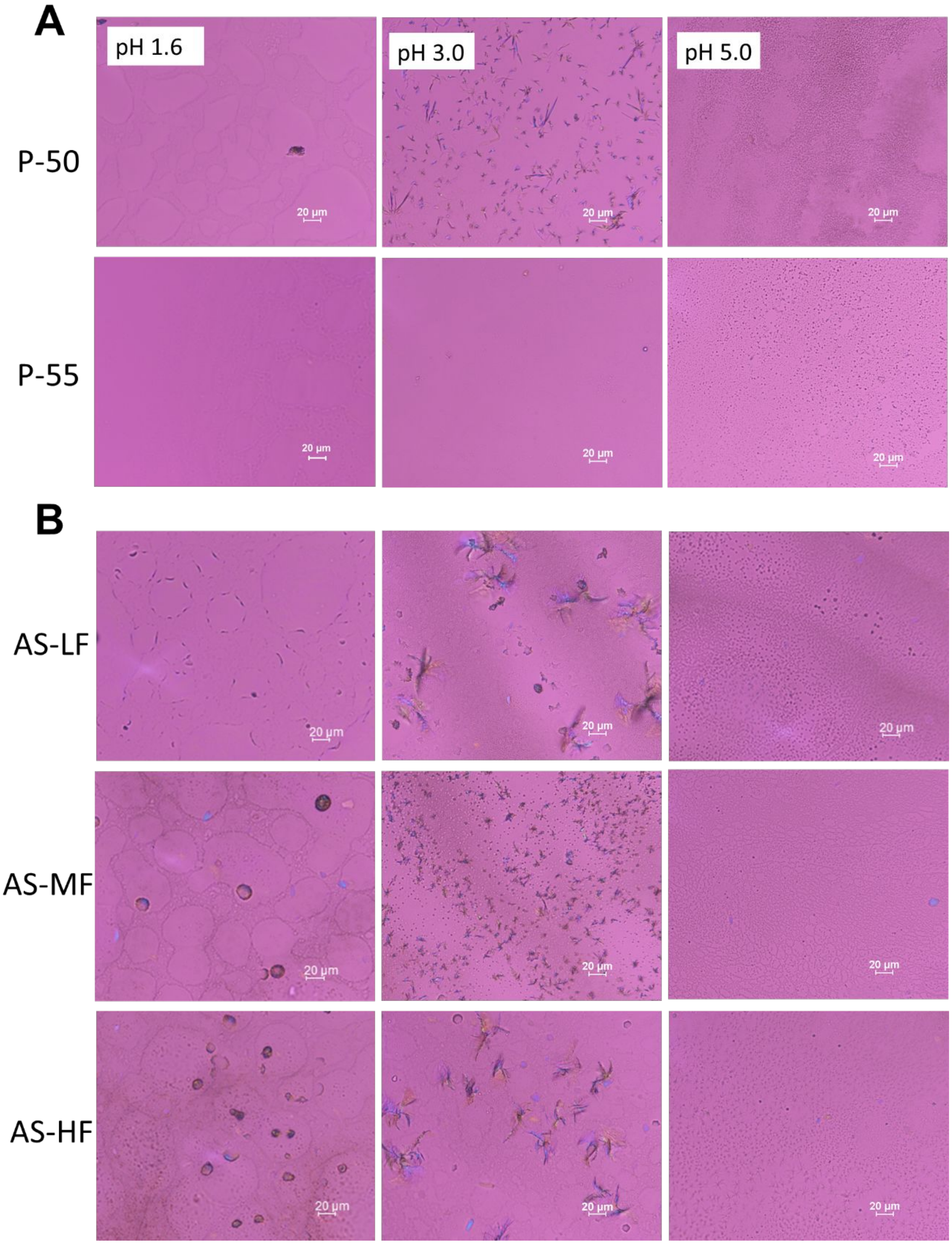

Figure S5. PLM images showing drug crystallization from DLM edisylate ASD films with (A) HPMCP and (B) HPMCAS after incubation in acidic environments for 1 h.

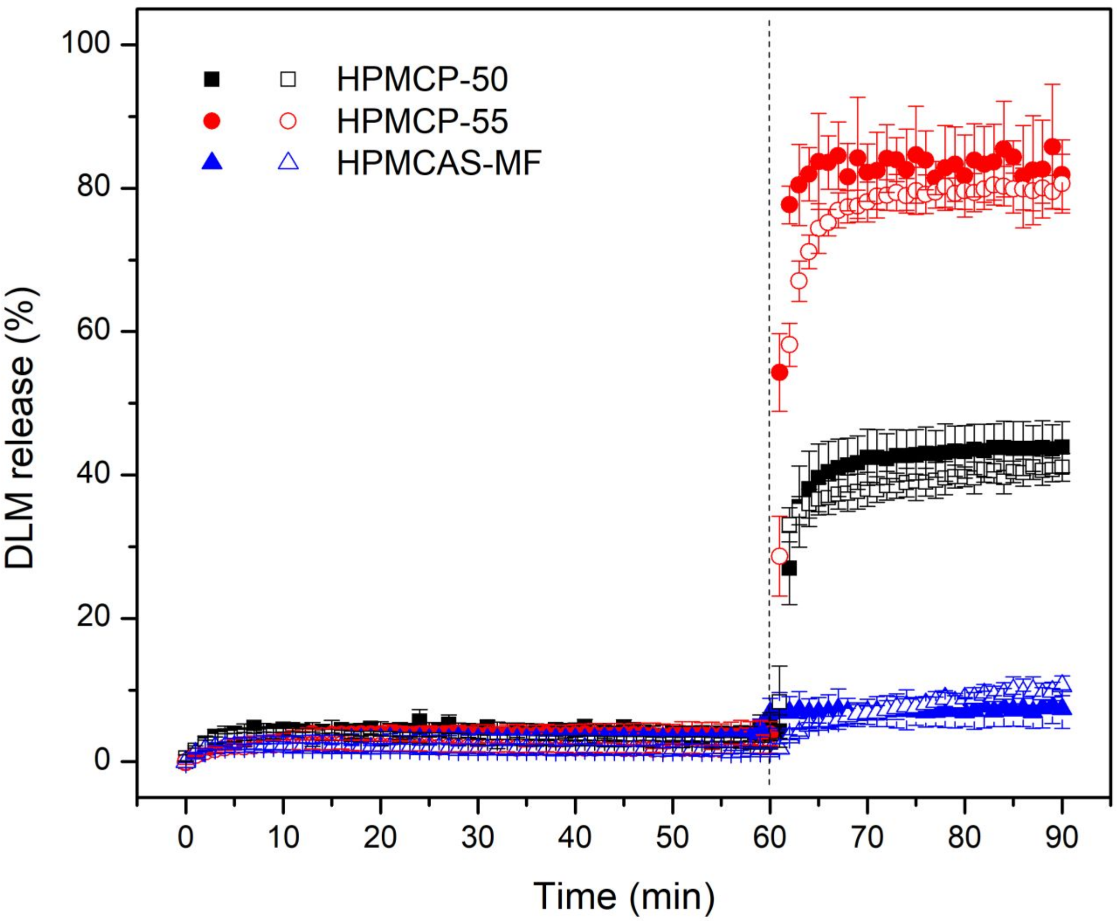

Figure S6: Drug release from ASDs of DLM-edisylate (25% DL) with HPMCP-50, HPMCP-55 and HPMCAS-MF in phosphate buffer pH 3.0 to pH 6.5 with (solid symbol) and without (open symbol) pre-dissolved PVPVA (1 mg/mL) as a solution drug crystallization inhibitor.

References

1. Wang, S.; Liu, C.; Chen, Y.; Zhang, Z.; Zhu, A.; Qian, F., A high-sensitivity HPLC-ELSD method for HPMC-AS quantification and its application in elucidating the release mechanism of HPMC-AS based amorphous solid dispersions. *Eur. J. Pharm. Sci.* **2018,** *122*, 303-310.

2. Rashan, J.; Chen, R., Developing a versatile gradient elution LC/ELSD method for analyzing cellulose derivatives in pharmaceutical formulations. *J. Pharm. Biomed. Anal.* **2007,** *44* (1), 23-28.
